# Positively Charged Pt‐Based Nanoreactor for Efficient and Stable Hydrogen Evolution

**DOI:** 10.1002/advs.202203199

**Published:** 2022-08-09

**Authors:** Kun Feng, Jiabin Xu, Yufeng Chen, Shuo Li, Zhenhui Kang, Jun Zhong

**Affiliations:** ^1^ Institute of Functional Nano and Soft Materials Laboratory (FUNSOM) Jiangsu Key Laboratory for Carbon‐Based Functional Materials & Devices Soochow University Suzhou 215123 P. R. China; ^2^ Department of Chemistry University of Western Ontario London Ontario N6A 5B7 Canada; ^3^ Macao Institute of Materials Science and Engineering (MIMSE) MUST‐SUDA Joint Research Center for Advanced Functional Materials Macau University of Science and Technology Taipa Macao 999078 P. R. China

**Keywords:** hydrogen evolution reaction, nanoreactors, positively charged Pt, X‐ray absorption spectroscopy

## Abstract

Positively charged Pt can work as the active center for hydrogen evolution reaction (HER) but the corresponding design of state‐of‐the‐art electrocatalysts at high current densities has never been realized. Here the application of positively charged Pt in an effective Fe‐PtNiPO nanoreactor for highly efficient and stable HER is demonstrated. Synchrotron radiation X‐ray absorption spectroscopy confirms the formation of internal positively charged Pt and the in situ experiments reveal the quick charge transfer in the nanoreactor. Ni‐based materials around Pt are used to tune the electronic structure and promote the water dissociation to form locally enriched H^+^, while a porous Fe shell can both prevent the loss of active material and allow the efficient material transport. All the beneficial compositions work together to form an effective nanoreactor for HER. As a result, the Fe‐PtNiPO nanoreactor shows a low overpotential of 19 mV to achieve 10 mA cm^−2^ and exhibits a high mass activity of 10.93 A mg_Pt_^−1^ (at 100 mV). Most importantly, it only needs an ultra‐low overpotential of 193 mV to achieve a high current density of 1000 mA cm^−2^ with an excellent stability over 300 h, which represents one of the best electrocatalysts for alkaline HER and might be used for large‐scale industrial application in the future.

## Graphical abstract

Positively charged Pt is created in a nanoreactor as the active center for highly efficient and stable alkaline hydrogen evolution reaction (HER), where the surrounding Ni can tune the electronic structure and promote the water dissociation, and the outside porous Fe shell can both prevent the loss of active material and allow the efficient material transport.

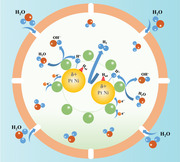

## 1 Introduction

Electrochemical hydrogen evolution reaction (HER) has been widely studied for producing clean hydrogen from water.^[^ ^1^, ^2^, ^3^, ^4^, ^5^, ^6^, ^7^, ^8^ ^]^ Unfortunately, the practical HER performance is highly limited by the lack of efficient and stable electrocatalysts, especially those at high currents over 500 mA cm^−2^ for industrial application.^[^ ^9^, ^10^, ^11^, ^12^ ^]^ Pt‐based materials are excellent HER electrocatalysts and Pt metal (Pt^0^) has been typically considered as the active center.^[^ ^13^, ^14^, ^15^, ^16^ ^]^ However, some recent reports revealed that the presence of oxygen in Pt‐based catalysts could lead to superior HER performance than pure Pt metal.^[^ ^17^, ^18^ ^]^ Moreover, the positively charged Pt (with the loss of electrons, including but not limited to oxidized Pt) was proved to be the real catalytic center, which could accelerate the coupling of proton and electron and then lead to rapid release of H_2_ for excellent HER performance.^[^ ^19^ ^]^ The positively charged Pt was further studied by exploring the coordination environment of Pt and evaluating the charge transfer capability for better HER activity.^[^ ^20^ ^]^ Although the working mechanism of positively charged Pt as active center has been investigated, unfortunately, the corresponding design of state‐of‐the‐art electrocatalysts based on positively charged Pt for efficient and stable HER, especially those at high working currents, has never been realized until now.

Inspired by the above mechanism studies, here we demonstrated the application of positively charged Pt in an effective nanoreactor for efficient and stable HER in alkaline medium. Synchrotron radiation‐based X‐ray absorption spectroscopy (XAS) strongly confirms the formation of positively charged Pt in the Fe‐PtNiPO nanoreactor, while the in situ XAS experiments reveal the quick charge transfer from the electrode to the internal Pt sites. As a designed nanoreactor, Ni‐based materials around the Pt sites have been used to tune the electronic structure and promote the water dissociation to form locally enriched H^+^ in alkaline solution.^[^ ^6^, ^15^, ^21^, ^22^, ^23^, ^24^, ^25^ ^]^ Moreover, to protect the inside active sites with a “chainmail catalyst” effect,^[^ ^26^, ^27^ ^]^ here by a simple annealing and acid washing process, a favorable porous Fe shell can be created to both prevent the loss of active material and allow the efficient material transport. As a result, the Fe‐PtNiPO nanoreactor with internal positively charged Pt sites shows a low overpotential of 19 mV to obtain 10 mA cm^−2^ for alkaline HER and exhibits a high mass activity of 10.93 A mg_Pt_^−1^ (at 100 mV). Most importantly, it only needs an ultralow overpotential of 193 mV to achieve the large current of 1000 mA cm^−2^, with an excellent stability over 300 h. To the best of our knowledge, it may represent one of the best HER catalysts ever reported for large working currents, which can fully meet the requirements for large‐scale industrial applications.

## 2 Results

### 2.1 Morphology and Structure Characterization

**Figure 1:**
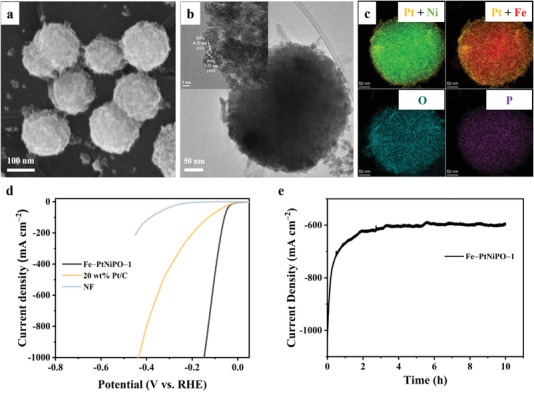
Morphology information and HER performance of Fe‐PtNiPO‐1. a) SEM image, b) HRTEM image and c) the corresponding dark‐field elemental mappings of Fe‐PtNiPO‐1: Fe (red), Ni (green), Pt (yellow), P (purple) and O (cyan). d) HER polarization curves (after 90% i*R* compensation) of Fe‐PtNiPO‐1, 20 wt% Pt/C and NF, respectively. e) Stability test of Fe‐PtNiPO‐1 by keeping the overpotential at 150 mV (after 90% i*R* compensation) for 10 h (with an initial current density of 1000 mA cm^−2^).

A two‐step electrodeposition method was used to synthesize the initial Fe‐PtNiPO catalyst. First, FeNiPO microspheres were directly grown on nickel foam (NF). Second, Pt was dispersed on these spheres via a linear sweep voltammetry method (Fe‐PtNiPO‐1 sample). The detailed synthesis information can be found in Supporting Information. X‐ray diffraction (XRD) data of the prepared samples are shown in Figure S1 (Supporting Information). The major sharp peaks at about 44°, 52°, and 76° can be attributed to Ni foam (JCPDS No. 04‐0850) without any other features, which might be attributed to the very low loading content. Scanning electron microscope (SEM) image of Fe‐PtNiPO‐1 is shown in **Figure** **1**a, revealing the microsphere structure with a rough surface (diameter around several hundred nm). SEM images of the control samples with the absence of some specific elements (such as Pt, Ni, P, and Fe) are also shown in Figure S2 (Supporting Information). Interestingly, the samples without Pt (Fe‐NiPO) or Fe (PtNiPO) are still microspheres, while the samples without Ni (Fe‐PtPO) or P (Fe‐PtNiO) cannot maintain the favorable sphere morphology. The corresponding HER curves of the samples are also shown in Figure S2e (Supporting Information). Without Pt, the performance is very poor suggesting that Pt is the active site for HER. Moreover, the absence of any other elements (Fe, Ni, or P) will also lead to a decreased HER performance, suggesting the favorable composition and morphology of the present catalyst. Especially, the P content in Fe‐PtNiPO‐1 is ultra‐low (0.02 wt%) but the HER performance for Fe‐PtNiO (without P) decreases a lot, which might be assigned to the morphology change (the loss of sphere structure).

Transmission electron microscope (TEM) images and the corresponding elemental mappings of Fe‐PtNiPO‐1 are shown in Figure 1b,c, respectively. The microsphere structure can be clearly identified. All the elements can be observed in the mappings with an outside Pt layer. The contents of Pt, Fe, and P in the samples are probed by inductively coupled plasma optical emission spectrometer (ICP‐OES) and the results are shown in Table S1 (Supporting Information). The major content (more than 90%) measured in the sample is Ni from Ni foam. P was reported to increase the catalytic performance.^[^ ^16^, ^22^ ^]^ However, here the P content is very low and it mainly plays an important role to form the sphere structure (Figure S2, Supporting Information). X‐ray photoelectron spectroscopy (XPS) spectra of Fe‐PtNiPO‐1 are also shown in Figure S3 (Supporting Information). Ni 2p spectrum shows a main peak at 856.4 eV for Ni 2p_3/2_, indicating the presence of Ni(OH)_2_.^[^ ^28^ ^]^ Fe 2p spectrum suggests the presence of Fe^3+^ (similar to Fe_2_O_3_) while Pt 4f spectrum suggests the presence of Pt metal with a small amount of Pt^II^.

The HER activity of Fe‐PtNiPO‐1 is shown in Figure 1d. Interestingly, the catalyst shows a superior catalytic activity, which is much higher than that of the commercial Pt/C catalyst but with less Pt content (Table S1, Supporting Information). The Fe‐PtNiPO‐1 only requires an overpotential of 19 mV to achieve 10 mA cm^−2^ and shows great advantage at high current densities (> 500 mA cm^−2^).^[^ ^29^ ^]^ It can be attributed to the favorable structure, which exhibits a sphere morphology with highly dispersed Pt layer. The outside Pt can catalyze the HER reaction with the assistance of Ni‐based sphere. Pt‐based materials are excellent HER catalysts but their activities have been strongly limited by the low concentration of H^+^ in alkaline medium. To solve this problem, Ni(OH)_2_ particles were first added on Pt surface to promote the water dissociation, which could create a favorable local environment with enriched H^+^ to accelerate the HER process.^[^ ^6^ ^]^ Since then, various Ni‐based materials have been coupled with Pt to promote the alkaline HER activity.^[^ ^21^, ^22^, ^23^ ^]^ In this work, the outside Pt in Fe‐PtNiPO‐1 can act as the active center to accelerate the coupling of proton and electron and lead to rapid release of H_2_, while the surrounding Ni in the sphere can promote the water dissociation for better HER performance in alkaline solution.^[^ ^6^ ^]^ Due to the abundant Pt–Ni interface,^[^ ^21^ ^]^ the HER performance can thus be greatly enhanced when compared to the Pt/C electrode. Unfortunately, the highly efficient Fe‐PtNiPO‐1 catalyst shows an unstable performance (keeping the overpotential at 150 mV for 10 h), especially at high currents, as shown in Figure 1e (decrease in a few minutes). Similar stability problem was also reported in many other Pt‐based catalysts with high catalytic activity.^[^ ^30^, ^31^ ^]^

**Scheme 1:**
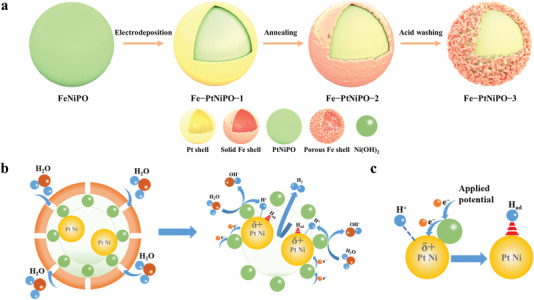
Schematic illustration for the Fe‐PtNiPO catalysts. a) The preparation of Fe‐PtNiPO‐1, Fe‐PtNiPO‐2, and Fe‐PtNiPO‐3. b) Working mechanism for the Fe‐PtNiPO‐3 nanoreactor. c) The formation of H_ad_ on the surface of Fe‐PtNiPO‐3.

To solve this problem, we have created a porous shell by a simple annealing and acid washing process to both protect the active catalyst and allow the reactant transport (illustrated in **Scheme** **1**a). As shown in Figure S4 (Supporting Information), the sample after annealing (Fe‐PtNiPO‐2) can maintain the sphere morphology. However, Fe will migrate from the inner core to form an outside protection shell, while Pt and Ni stay inside as the active core. Similar phenomenon with a Fe shell can also be observed for the sample without Pt (Figure S5, Supporting Information). With a protection layer, the reaction stability can thus be significantly improved as shown in Figure S6 (Supporting Information). However, the solid Fe layer after annealing will block the material transport and the HER activity of Fe‐PtNiPO‐2 sharply decreases, showing an overpotential of 182 mV to achieve 10 mA cm^−2^ in Figure S6 (Supporting Information).

**Figure 2:**
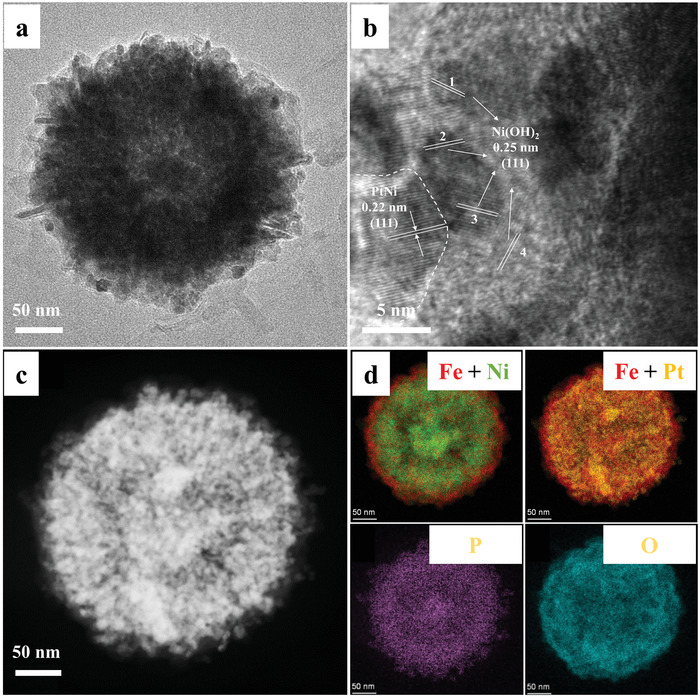
Morphology information of Fe‐PtNiPO‐3. a) TEM image, b) HRTEM image, c) HAADF‐STEM image and d) the corresponding dark‐field elemental mappings of Fe‐PtNiPO‐3: Fe (red), Ni (green), Pt (yellow), P (purple) and O (cyan).

In order to realize both high catalytic activity and good stability, we further treated the sample (Fe‐PtNiPO‐3) by acid washing to form a porous shell for effective reactant transport (Scheme 1). As shown in **Figure** **2**, the Fe‐PtNiPO‐3 sample can maintain the sphere structure with the surface Fe‐based shell (Figure 2a,d). However, the outside Fe‐based shell shows an increased roughness with large amounts of porous structures to allow the material transport (Figure 2a). HRTEM image in Figure 2b further reveals the inner compositions in Fe‐PtNiPO‐3. Clear lattice structure (0.22 nm) for the (111) facet of PtNi alloy can be observed, with the surrounding crystal structure (0.25 nm) for the (111) facet of Ni(OH)_2_.^[^ ^32^, ^33^ ^]^ The contents of Pt, Fe, and P in various Fe‐PtNiPO samples are shown in Table S1 (Supporting Information), in which the three Fe‐PtNiPO samples show similar contents of Pt, Fe, and P. According to the XPS spectra in Figure S7 (Supporting Information), Fe is oxidized while Pt in Fe‐PtNiPO‐3 is mainly metallic with a small amount of Pt^II^, suggesting the presence of positively charged Pt. The Fe and Pt results are similar to those for Fe‐PtNiPO‐1 and Fe‐PtNiPO‐2. Interestingly, Ni in Fe‐PtNiPO‐1 mainly exists as Ni(OH)_2_ according to the energy position, while Ni in Fe‐PtNiPO‐2 and Fe‐PtNiPO‐3 exhibits an obvious energy shift (Figure S7c, Supporting Information), suggesting the formation of NiO.^[^ ^21^ ^]^

### 2.2 Electrocatalytic HER Activity

**Figure 3:**
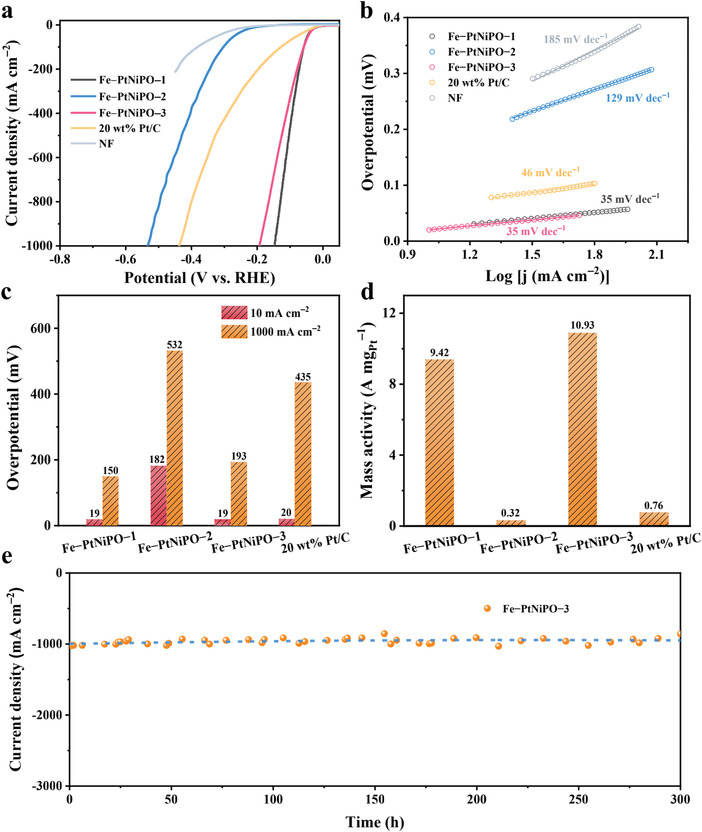
Electrocatalytic performance of different catalysts. a) HER polarization curves and b) Tafel curves (after 90% i*R* compensation) of the Fe‐PtNiPO samples, 20 wt% Pt/C and NF. c) Overpotentials at the current densities of 10 and 100 mA cm^−2^ and d) Pt‐based mass activities (at 0.1 V) of the Fe‐PtNiPO samples and 20 wt% Pt/C. e) Stability test of Fe‐PtNiPO‐3 by keeping the overpotential at 193 mV (after 90% i*R* compensation) for 300 h (with an initial current density of 1000 mA cm^−2^).

The electrocatalytic HER activities of Fe‐PtNiPO samples were evaluated by using a standard three‐electrode cell in 1.0 m N_2_‐saturated KOH. The catalysts on nickel foam (NF) were cut into 0.5 × 0.5 cm^2^ and then directly used as the working electrodes. All measurements were performed at 5 mV s^−1^ after 90% i*R* compensation at 25 °C except for the specific case with explanation. The electrocatalytic HER curves of Fe‐PtNiPO samples and some reference samples (20 wt% Pt/C and NF) are shown in **Figure** **3**a. Pure NF substrate shows a very large overpotential to achieve a current density of 10 mA cm^−2^, indicating that it is not an efficient HER catalyst and mainly works as a good support material. The commercial 20 wt% Pt/C catalyst shows a good alkaline HER performance with an overpotential of 20 mV to achieve 10 mA cm^−2^, in good agreement with the reported values.^[^ ^34^, ^35^ ^]^

As clearly demonstrated in Figure 3a, the final Fe‐PtNiPO‐3 sample with multiple channels on the Fe shell shows both excellent HER performance and good stability. For comparison, Fe‐PtNiPO‐2 with a solid Fe‐based shell shows a much lower HER performance than that of Fe‐PtNiPO‐3, confirming that the inside Pt and Ni‐based materials are the real active sites. Fe‐PtNiPO‐3 shows an excellent catalytic activity with an overpotential of only 19 mV for 10 mA cm^−2^, which is the same to that of Fe‐PtNiPO‐1 (Figure 3a,c). Moreover, at a high current density of 1000 mA cm^−2^ for the practical use, Fe‐PtNiPO‐3 exhibits a much lower overpotential (193 mV) than that of the commercial 20 wt% Pt/C (435 mV) and Fe‐PtNiPO‐2 (532 mV). For comparison, by now Raney Ni is used for industrial alkaline HER at high current densities, but it requires high overpotentials of ≈300−500 mV to achieve 500 mA cm^−2^ and shows high Tafel slopes of ≈90−120 mV dec^−1^ in even 30 wt% KOH solution.^[^ ^9^, ^10^ ^]^ Although the HER activity of Fe‐PtNiPO‐3 is slightly lower than Fe‐PtNiPO‐1 at high current densities, it simultaneously exhibits a good stability along with the high efficiency. As shown in Figure 3e, Fe–PtNiPO‐3 can maintain a high current density of 1000 mA cm^−2^ (keeping the overpotential at 193 mV) for more than 300 h without distinct activity loss, indicating an industrial level catalytic stability. Figure S8b (Supporting Information) also shows the polarization curves of Fe‐PtNiPO‐3 before and after 2000 cyclic voltammetry (CV) sweeps in alkaline HER, which exhibit almost no change. The curves of Fe‐PtNiPO‐1 before and after 2000 cycles are also shown in Figure S8a (Supporting Information) for comparison. The high catalytic activity with excellent stability represents one of the best alkaline HER performance ever reported at high current densities (a comparison to the literature can be found in Table S2, Supporting Information). The prominent catalytic activity of Fe‐PtNiPO‐3 is also consistent with its very low Tafel slope of 35 mV dec^−1^ in Figure 3b, as compared with those of Fe‐PtNiPO‐2 (129 mV dec^−1^), 20 wt% Pt/C (46 mV dec^−1^) and NF (185 mV dec^−1^), indicating the better HER kinetics.^[^ ^15^, ^36^ ^]^ The Tafel slope for Fe‐PtNiPO‐1 is almost the same as that for Fe‐PtNiPO‐3, consistent with its high catalytic activity (unfortunately with low stability). A detailed comparison of the overpotentials at 10 and 1000 mA cm^−2^ for various catalysts can also be found in Figure 3c, confirming the high performance of Fe‐PtNiPO‐3.

Considering the high cost of noble‐metal elements, the mass activity is also an important issue to evaluate the catalysts. The Pt content in Fe‐PtNiPO‐3 is only around 0.09% (Table S1, Supporting Information), which is much lower than the commercial 20 wt% Pt/C catalyst (0.29 wt% Pt in the electrode). As shown in Figure 3d, both Fe‐PtNiPO‐1 and Fe‐PtNiPO‐3 exhibit much higher mass activities than that of the commercial 20 wt% Pt/C. Especially, Fe‐PtNiPO‐3 shows a mass activity of 10.93 A mg_Pt_^−1^ at the overpotential of 0.1 V, which is 14 times higher than that of 20 wt% Pt/C, confirming the wonderful improvement with the current design of Pt‐based nanoreactor. The highly efficient and stable Fe‐PtNiPO‐3 catalyst with low Pt content might be used for practical alkaline water splitting in the future.

The electrode kinetics and interfacial properties of Fe‐PtNiPO‐3 were elucidated by electrochemical impedance spectroscopy (EIS). After fitting the Nyquist plots (Figure S9, Supporting Information), the solution resistance (*R*_s_) values and the charge transfer resistance (*R*_ct_) values can be obtained (Table S3, Supporting Information). The *R*_s_ values have been applied to correct the polarization curves with i*R* compensation. Fe‐PtNiPO‐3 shows a much lower *R*_ct_ of 4.3 Ω than that of 20 wt% Pt/C, suggesting a faster charge transfer from the electrode to electrolyte for better HER performance. Although the *R*_s_ and *R*_ct_ values for Fe‐PtNiPO‐1 are even lower than that for Fe‐PtNiPO‐3 standing for higher catalytic activity, the poor stability of Fe‐PtNiPO‐1 strongly limits its application. The CV curves at different scan rates and the calculated double‐layer capacitance (*C*_dl_) for various catalysts can also be found in Figure S10 (Supporting Information). Fe‐PtNiPO‐3 shows a higher *C*_dl_ value of 16.0 mF cm^−2^ than that of Fe‐PtNiPO‐2 (11.3 mF cm^−2^) and 20 wt% Pt/C (10.3 mF cm^−2^), suggesting more active sites. It shows a lower *C*_dl_ value than that of Fe‐PtNiPO‐1 (20.2 mF cm^−2^), which can be attributed to the inert Fe shell which will partially block the active sites.

### 2.3 Synchrotron Radiation Study and Working Mechanism

**Figure 4:**
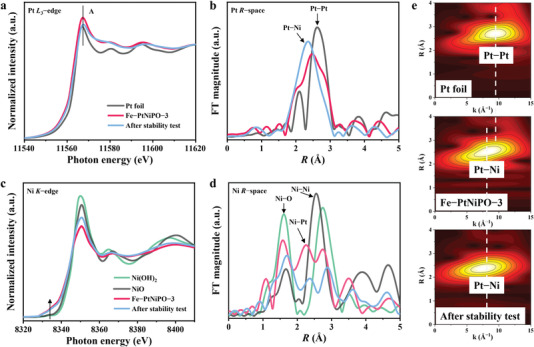
Structural identification of Fe‐PtNiPO‐3. XANES spectra of Fe‐PtNiPO‐3 before and after stability test at a) Pt *L*_3_‐edge and c) Ni *K*‐edge, respectively. b,d) The corresponding Fourier transform curves of the EXAFS data. The reference spectra of Pt foil, NiO, and Ni(OH)_2_ are also shown for comparison. e) WT plots of the EXAFS data for Pt foil, Fe‐PtNiPO‐3, and Fe‐PtNiPO‐3 (after stability test), showing the *k* and *R* space data simultaneously.

Synchrotron radiation X‐ray absorption spectroscopy has been used to probe the working mechanism. Figure S11 (Supporting Information) shows the XAS spectra of Fe‐PtNiPO‐1 and Fe‐PtNiPO‐2. In Figure S11a (Supporting Information), the white line peak (peak A) intensities of both Fe‐PtNiPO‐1 and Fe‐PtNiPO‐2 are obviously higher than that of Pt foil, strongly suggesting the formation of positively charged Pt. Moreover, the Fe‐PtNiPO‐2 sample shows an increased shoulder than that of Fe‐PtNiPO‐1 in the X‐ray absorption near‐edge structure (XANES) spectrum, revealing more positive charge after annealing. It should be noted that the positively charged Pt means the loss of electrons from the outer‐most orbitals of Pt, which might come from both a formal oxidation or an interfacial interaction between Pt and the oxidative environment. After acid washing, Fe‐PtNiPO‐3 in **Figure** **4**a also exhibits a XANES spectrum similar to that of Pt foil but the white line peak intensity obviously increases, confirming the presence of positively charged Pt. The corresponding Fourier transform (FT) curves of extended X‐ray absorption fine‐structure (EXAFS) data are shown in Figure 4b. Pt foil shows a main peak at around 2.6 Å for Pt–Pt bonds, while Fe‐PtNiPO‐3 shows a main peak at about 2.4 Å with a shorter radial distance, suggesting the existence of Pt‐Ni coordination.^[^ ^37^, ^38^, ^39^ ^]^ The result clearly reveals the existence of PtNi alloy in Fe‐PtNiPO‐3, in good agreement with the HRTEM results. After stability test, the Fe‐PtNiPO‐3 sample shows a similar Pt–Ni coordination in Figure 4b, suggesting the stable internal structure. The Fe‐PtNiPO‐1 and Fe‐PtNiPO‐2 samples also show the prominent Pt–Ni coordination in Figure S11b (Supporting Information). Fe‐PtNiPO‐1 shows a lower peak intensity than Fe‐PtNiPO‐2 which might be attributed to less contact between Pt and Ni before annealing. More evidence for the Pt‐Ni coordination can be found in the wavelet transform (WT) plots of Pt in Figure 4e. The WT plots are used to intuitively display both *k*‐space and *R*‐space data in a 2D way to discriminate the backscattering atoms.^[^ ^40^ ^]^ The heavier atom (high atomic number) has a strong ability to scatter photoelectrons, thus its strongest oscillation will appear at higher frequencies (high *k* values).^[^ ^41^ ^]^ For Pt foil, Pt–Pt peak exists at a *k* value of ≈10 Å^−1^ while for Fe‐PtNiPO‐3, the main peak appears at a lower *k* value (≈8.5 Å^−1^) confirming the existence of Pt–Ni coordination. The WT plots for Fe‐PtNiPO‐1 and Fe‐PtNiPO‐2 have also been shown in Figure S12 (Supporting Information), which show the similar results. EXAFS fitting at Pt *L*_3_‐edge has also been shown in Figure S13 (Supporting Information) to probe the coordination environment. The corresponding parameters are listed in Table S4 (Supporting Information). The fitting results confirm the presence of Pt–Ni bonds with some weak Pt–O bonds in the catalysts. The presence of Pt–Ni bonds can lead to the formation of positively charged Pt and partly reduced Ni in the Fe‐PtNiPO samples, which could accelerate the coupling of proton and electron and lead to rapid release of H_2_. The XAS spectra for Fe‐PtNiPO‐3 after stability test are also shown in Figure 4b at Pt *L*_3_‐edge, in which the peak for Pt–Ni coordination is still a main feature suggesting the excellent structural stability. Actually, as shown in Table S1 (Supporting Information), the content of active Pt shows almost no change after long‐term stability test, confirming the excellent stability of Fe‐PtNiPO‐3. The XRD pattern of Fe‐PtNiPO‐3 after stability test is also shown in Figure S1 (Supporting Information), while no new diffraction peaks can be observed.

The XANES spectra of Fe‐PtNiPO samples at Ni *K*‐edge with Ni(OH)_2_ and NiO as the reference samples are also shown in Figure 4c and Figure S11c (Supporting Information). All the Fe‐PtNiPO samples show an increased pre‐peak (indicated by arrow in Figure 4c) when compared to Ni(OH)_2_ or NiO, revealing the partly reduced state of Ni with the presence of Pt. It is consistent with the formation of positively charged Pt in Figure 4a. From the EXAFS data in Figure S11d (Supporting Information), Fe‐PtNiPO‐1 shows two main peaks for Ni–O bond and Ni–Ni coordination, respectively. The peak position for Ni–Ni (at 2.73 Å) is similar to that for Ni(OH)_2_, suggesting the main Ni(OH)_2_ content for Ni species in Fe‐PtNiPO‐1. After annealing in Ar, most of the Ni(OH)_2_ has been converted to NiO in Fe‐PtNiPO‐2, as revealed by the similar Ni‐Ni position (at 2.55 Å) as that for the NiO reference (Figure S11d, Supporting Information). Interestingly, after acid washing the Fe‐PtNiPO‐3 sample shows a Ni–Ni position similar to Ni(OH)_2_ again in Figure 4d, suggesting the easy transition of the present sample from NiO to Ni(OH)_2_ in solution. Moreover, a new peak at around 2.3 Å can also be observed, suggesting the formation of more PtNi alloy in Fe‐PtNiPO‐3. The results are in good agreement with the Pt *L*_3_‐edge XAS data and the TEM results. After long‐term HER reaction, the bonds for both PtNi alloy and Ni(OH)_2_ can still be clearly observed in Figure 4d suggesting the stable structure.

The XAS spectra at Fe *K*‐edge are shown in Figure S11 (Supporting Information). All the Fe‐PtNiPO samples before HER reaction show a high chemical state similar to that of Fe^3+^ in Fe_2_O_3_, as revealed by both XANES and EXAFS data. However, the sample after stability test shows a low chemical state similar to Fe foil suggesting the reduction of Fe shell in the HER process. The surface Fe may partly dissolve in the solution with the reduction, leading to a decreased content of Fe after the stability test as shown in Table S1 (Supporting Information) (from 0.31 to 0.21 wt%). Interestingly, the chemical state changes of Fe do not affect the HER performance as shown in Figure S8b (Supporting Information), suggesting that the Fe shell in Fe‐PtNiPO‐3 mainly acts as a stable shell to protect the active catalyst. The stability of such morphology in Fe‐PtNiPO‐3 has been illustrated by the corresponding TEM images (Figure S14, Supporting Information), suggesting the favorable morphology for highly efficient and stable HER. Thus in the Fe‐PtNiPO‐3 sphere, the P element help to form the sphere structure as revealed by Figure S2 (Supporting Information), the positively charged Pt acts as the active catalytic center, the Ni element in both PtNi alloy and Ni(OH)_2_ tunes the electronic structure of Pt and promotes the water dissociation to form locally enriched H^+^, and the porous Fe shell can both prevent the loss of active material and allow the efficient material transport. All the beneficial compositions work together to form an effective nanoreactor for the efficient and stable HER at high currents.

Since the surrounding environment of Pt also played an important role for the charge transfer from the electrode to the active Pt sites in HER,^[^ ^20^ ^]^ in situ XAS experiments at Pt *L*_3_‐edge (experimental setup shown in Figure S15, Supporting Information) were also used to test the charge transfer efficiency of the Fe‐PtNiPO‐3 nanoreactor. The Fe‐PtNiPO‐3 catalyst was electrodeposited on carbon paper as the electrode for in situ XAS experiments. External potentials were applied to the electrodes in the reaction process. The in situ XAS spectra are shown in Figure S16 (Supporting Information). The initial sample in air shows a higher white line peak intensity than that of Pt foil, suggesting the presence of positively charged Pt as that for the powder sample in Figure 4. Interestingly, even at an open‐circuit voltage, the white line peak of Pt quickly decreases to exhibit an intensity similar to that for Pt foil. The result suggests that the electrons can be quickly and efficiently transported from the electrode to the active Pt sites in Fe‐PtNiPO‐3, revealing the effective charge transfer in the nanoreactor. At a higher potential of 0.05 V versus RHE (bubbles can be observed), the spectrum will not change a lot, suggesting that the electrons have been fully used for the HER reaction at the active Pt sites. The working mechanism for Fe‐PtNiPO‐3 in alkaline HER can thus be established according to the above data, as illustrated in Scheme 1b,c. The porous Fe shell allows efficient material transport to the inside PtNi sites and the Ni‐based materials can tune the electronic structure of Pt and promote the water dissociation to form locally enriched H^+^. When external potentials are applied on the electrodes, electrons can be quickly transferred onto the positively charged Pt sites, which will work as the active catalyst to produce H_2_ with electrons and enriched H^+^, leading to an excellent HER performance. Moreover, the inert Fe shell can also protect the active materials in the nanoreactor to maintain a high stability.

## 3 Conclusions

To summarize, we have created an effective Pt‐based nanoreactor for efficient and stable alkaline HER. The positively charged Pt in the nanoreactor acts as the active catalytic center, the surrounding Ni‐based materials can tune the electronic structure and promote the water dissociation to form locally enriched H^+^, while the outside porous Fe shell of the nanoreactor will both prevent the loss of active material and allow the efficient material transport. As a result, the Fe‐PtNiPO‐3 nanoreactor has both superior catalytic activity and excellent stability, which exhibits a low overpotential of 19 mV to achieve 10 mA cm^−2^ and can work on a high current density of 1000 mA cm^−2^ at an ultra‐low overpotential of 193 mV for more than 300 h. It represents one of the best electrocatalysts ever reported for alkaline HER at high currents, which might be used for large‐scale industrial application in the future.

## Conflict of Interest

The authors declare no conflict of interest.

## Supporting information

Supporting Information

## Data Availability

The data that support the findings of this study are available from the corresponding author upon reasonable request.
