# Supplementary material for: Positively Charged Pt‐Based Nanoreactor for Efficient and Stable Hydrogen Evolution
Source: Adv Sci (Weinh). 2022 Aug 9;9(28):2203199. doi: 10.1002/advs.202203199 (PMC9534975; doi:10.1002/advs.202203199)
Supplement: Supplementary file 1 — Supporting Information [file ADVS-9-2203199-s001.pdf]

Supporting information

Positively charged Pt based nano-reactor for efficient and stable hydrogen evolution

Kun Feng[1†], Jiabin Xu[1,2†], Yufeng Chen[1], Shuo Li[1], Zhenhui Kang[1,3], and Jun Zhong[1]**

[1] Institute of Functional Nano and Soft Materials Laboratory (FUNSOM), Jiangsu Key Laboratory for Carbon-Based Functional Materials & Devices, Soochow University, Suzhou 215123, China

[2] Department of Chemistry, University of Western Ontario, London, Ontario N6A 5B7, Canada

[3] Macao Institute of Materials Science and Engineering, Macau University of Science and Technology, Taipa 999078, Macau SAR, China

[†] These authors contribute equally to this work.

E-mail: zhkang@suda.edu.cn; jzhong@suda.edu.cn

Methods

Chemicals and reagents. Ferrous sulfate heptahydrate ($FeSO_4·7H_2O$) was purchased from Sinopharm Chemical Reagent. Nickel Sulfate Hexahydrate ($NiSO_4·6H_2O$) was purchased from Enox. Chloroplatinic acid hexahydrate (H_2PtCl_6) was purchased from Aladdin. Sodium hypophosphite (NaH_2PO_2) was purchased from Sigma-Aldrich. Potassium hydroxide (KOH) was purchased from Macklin. Commercial Pt/C (20 wt%), Nafion (5 wt%) were purchased from Sigma-Aldrich. Carbon paper (TGP-H-060) was purchased from Japan Toray company. De-ionized water was obtained from an ultra-pure purifier (resistivity ⩾18.2 MΩ). All the reagents were of analytical grade and used as received without further purification.

Material preparation. Fe-PtNiPO samples were prepared by a two-step electrodeposition. Electrodeposition was conducted in a conventional three-electrode setup (CHI760E, Shanghai) with nickel foam (NF) as the working electrode, saturated calomel electrode (SCE) or Hg/HgO as the reference electrode, and Pt plate as the counter electrode. Before electrodeposition, nickel foam was washed by 5% HCl, acetone and distilled water in sequence via ultrasonic bath to remove surface oxide layer and contaminations. In the first step, nickel foam was immersed in the electrolyte containing 12.5 mM $FeSO_4·7H_2O$, 12.5 mM $NiSO_4·6H_2O$ and 0.5 M NaH_2PO_2 and then a potential of -0.95 V (vs. SCE) was applied for 30 min. Then nickel foam was transferred to 1 M KOH with 0.1 mM H_2PtCl_6 after washing with distilled water. The second step was carried out from -0.84 V to -1.34 V (vs. Hg/HgO) for cathodic deposition with a sweeping rate of 5 mV s^{-1}. This step was repeated for 15 times. After the deposition, the electrode was washed with distilled water again and dried under room temperature, then denoted as Fe-PtNiPO-1. Then it was transferred to a tube

furnace and heated at 400 °C under Ar atmosphere with the heating rate of 5 °C min^{-1}. Finally, after cooled down to room temperature, the sample (Fe-PtNiPO-2) was immersed in the 0.5 M H_2SO_4 for a few minutes, the obtained final sample was denoted as Fe-PtNiPO-3. Other reference samples, Fe-NiPO (without Pt), Fe-PtPO (without Ni), Fe-PtNiO (without P) and PtNiPO (without Fe) were prepared by the same synthesis process only without adding H_2PtCl_6, $NiSO_4·6H_2O$, NaH_2PO_2 and $FeSO_4·7H_2O$, respectively.

Structural Characterization. The morphology of the samples was measured by scanning electron microscopy (SEM, ZEISS G500) and high-resolution transmission electron microscopy (HRTEM, TALOS 200X). The metal contents were measured by an inductively coupled plasma (ICP) spectrometer (ICP-MS, Agilent 7700X). X-ray photoelectron spectrometer (XPS, Thermo Fischer ESCALAB 250X) and X-ray diffraction (XRD, Empyrean powder diffractometer) were used for structure characterization. X-ray absorption spectroscopy (XAS) experiments were performed at the Shanghai Synchrotron Radiation Facility (SSRF, 11B) and the National Synchrotron Radiation Laboratory (NSRL, Beamline MCD-B (Soochow Beamline for Energy Materials)). In order to eliminate the influence of NF, we have used carbon paper (CP) instead of NF for the same synthesis process to prepare the samples for XPS and XAS characterizations.

Electrochemical measurement. All electrochemical measurements were conducted by a standard three-electrode system (CHI 760E) at room temperature (~25 °C). 1.0 M KOH solution was used in all tests, with a Hg/HgO and a graphite rod as the reference and counter electrodes, respectively. The scan rate was 5 mV s^{-1} for linear sweep voltammetry (LSV) tests and 100 mV s^{-1} for long-term cyclic voltammetry (CV) tests. The potential has been converted to the value versus the

reversible hydrogen electrode (RHE) in this work, according to $E_{RHE} = E_{Hg/HgO} + 0.098 + 0.059 \times$ pH. The electrochemical impedance spectroscopy measurement (EIS) was carried out at an applied current density of 10 mA cm^{-2} over a frequency range from 0.1 Hz to 100 kHz with an additional alternating voltage at 5 mV. Electrochemically active surface area (ECSA) was estimated from the double layer capacitance (C_{dl}) charging curve using CV tests in a small potential range (-0.76 to -0.85 V *vs* Hg/HgO) with sweep rates from 20 to 100 mV s^{-1} at an interval of 20 mV. For high current density, a 90 % i*R* correction was taken. The stability tests were performed using the constant potential electrolysis method. All the catalysts were directly grown on Ni form as the working electrodes for electrochemical measurements. To prepare the Pt/C reference electrode, 20 wt% Pt/C (2 mg) was firstly dispersed in 1 ml Nafion solution (5 wt%) mixed with EtOH and water, and then sonicated for 30 min to form a homogeneous ink. Finally, 62.5 μl of the solution was loaded on Ni foam through drop-coating to form the electrode.

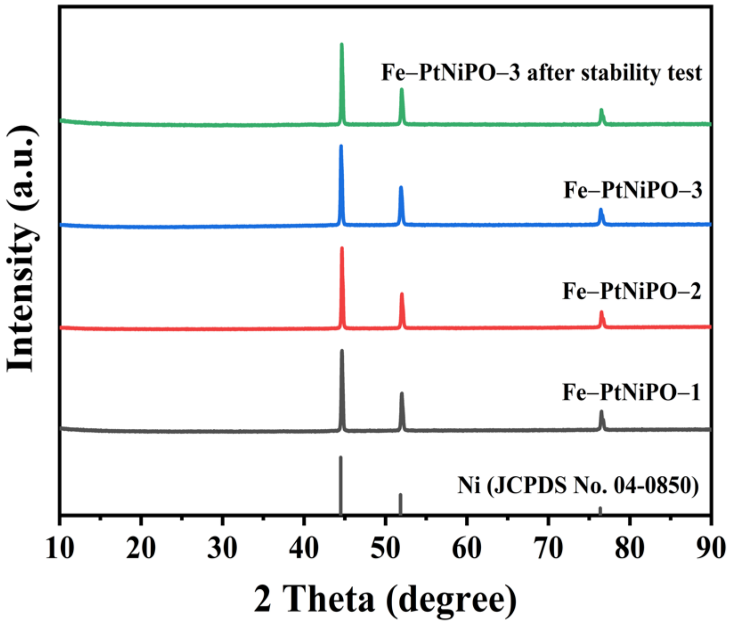

Figure S1. XRD patterns of various Fe-PtNiPO samples supported on nickel foam.

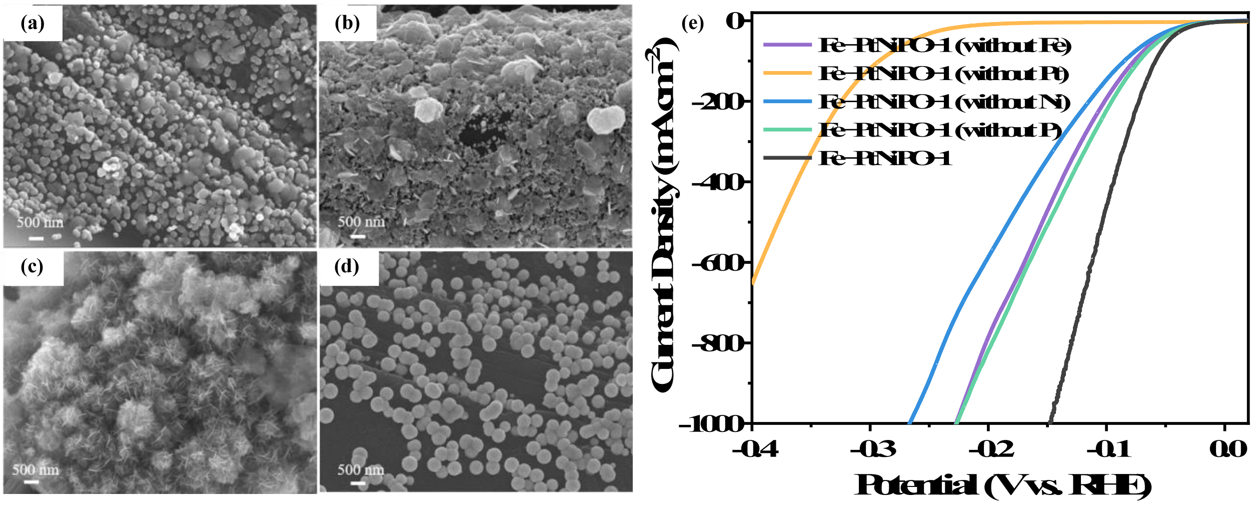

Figure S2. SEM images of (a) Fe-NiPO (without Pt), (b) Fe-PtPO (without Ni), (c) Fe-PtNiO (without P) and (d) PtNiPO (without Fe), respectively. (e) The corresponding HER polarization curves.

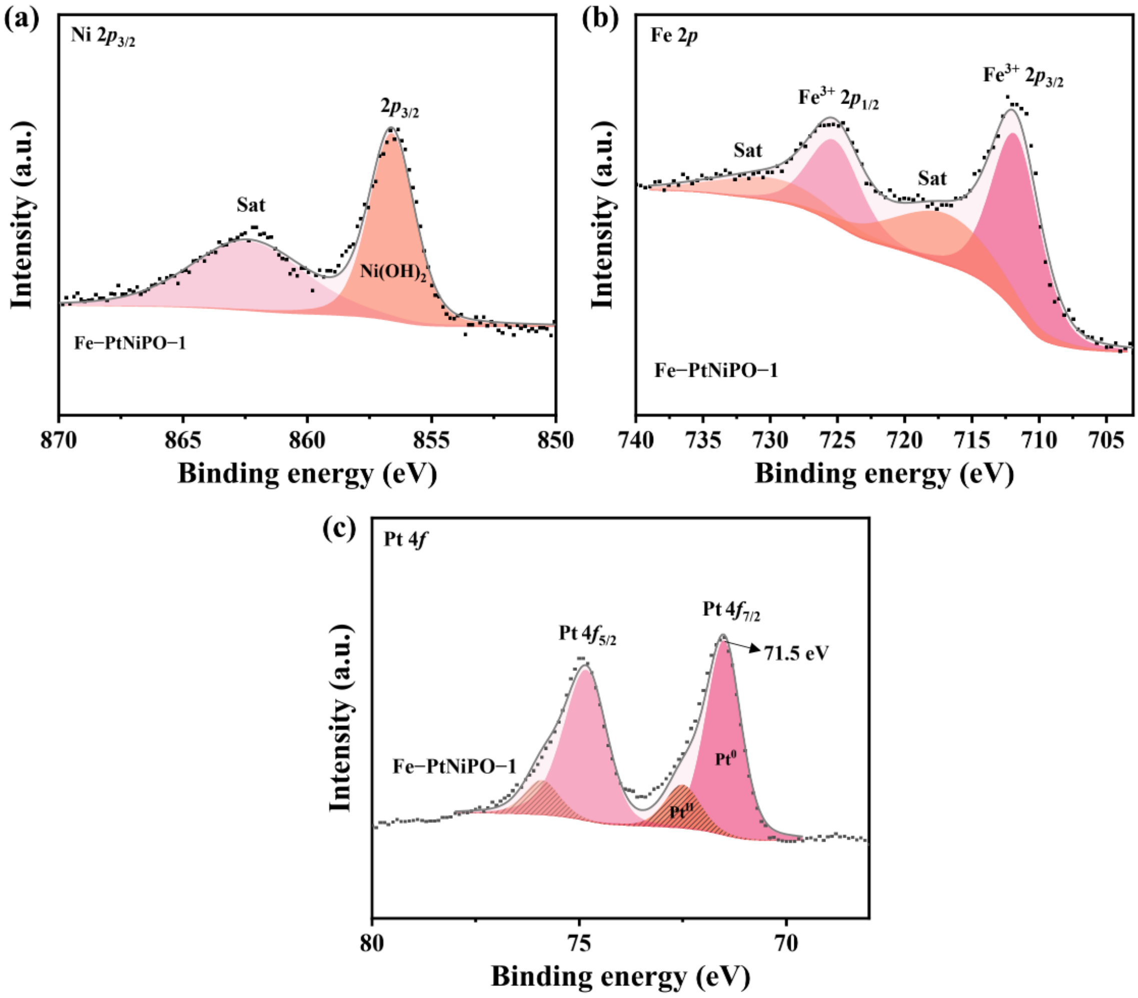

Figure S3. XPS spectra of Fe-PtNiPO-1 at (a) Ni $2p_{3/2}$, (b) Fe 2*p* and (c) Pt 4*f*, respectively.

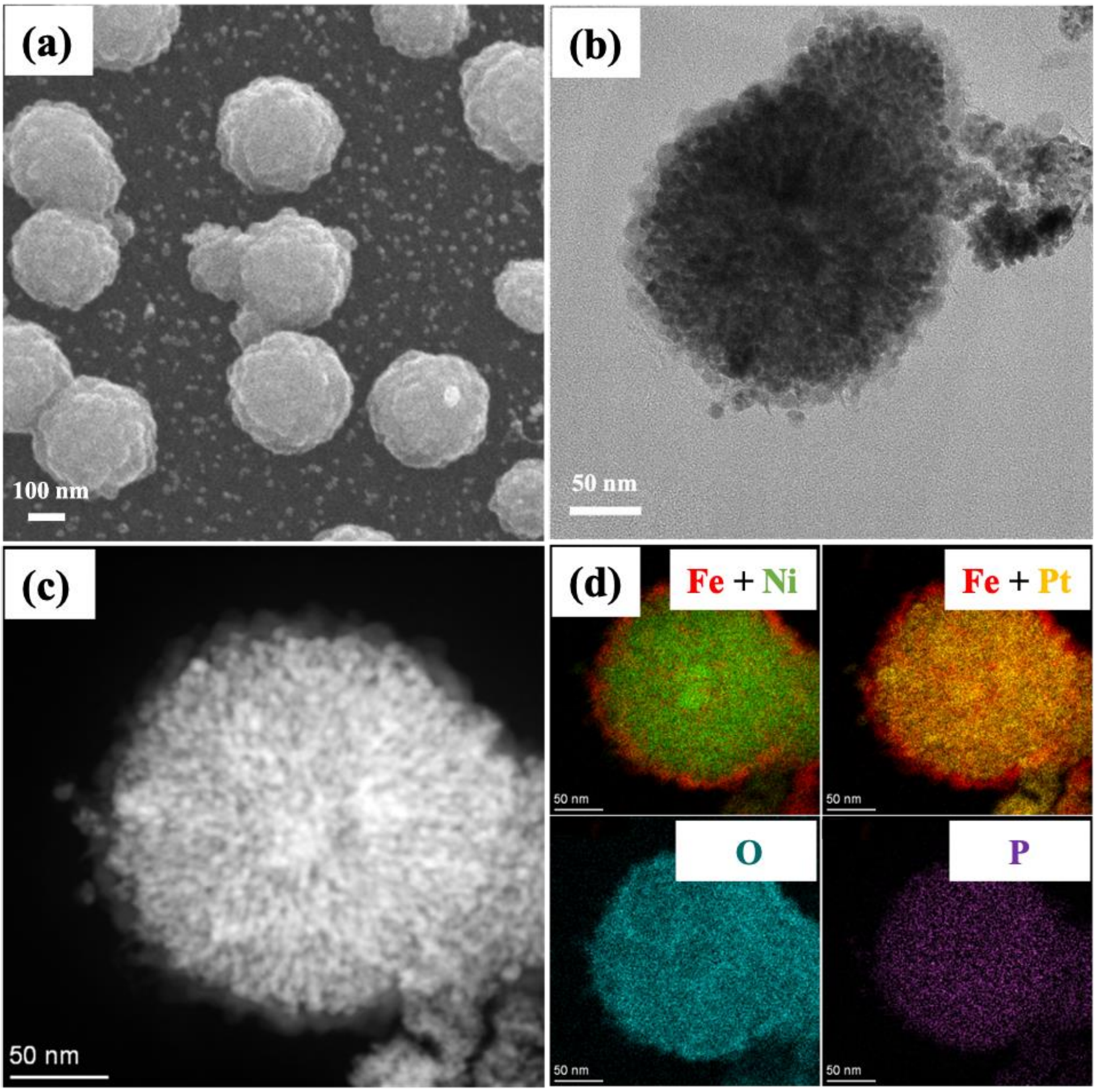

Figure S4. (a) SEM and (b) TEM images of Fe-PtNiPO-2, respectively. (c) HAADF-STEM image and (d) the corresponding dark-field elemental mappings of Fe-PtNiPO-2: Fe (red), Ni (green), Pt (yellow), P (purple) and O (cyan).

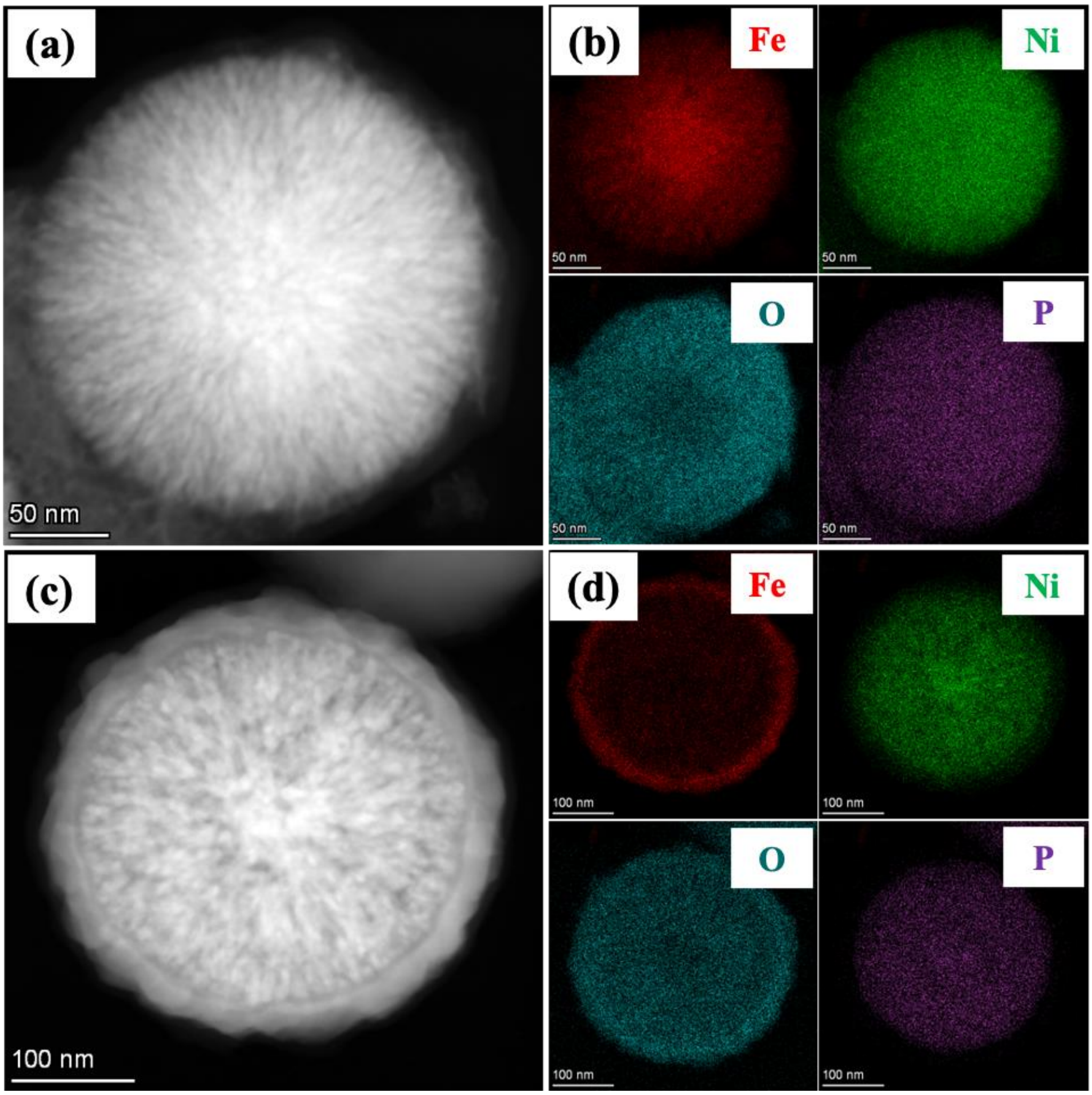

Figure S5. (a) HAADF-STEM image and (b) the corresponding dark-field elemental mappings of Fe-NiPO (before annealing). (c) HAADF-STEM image and (d) the corresponding dark-field elemental mappings of Fe-NiPO (after annealing). Fe, Ni, P and O are labeled as red, green, purple and cyan, respectively.

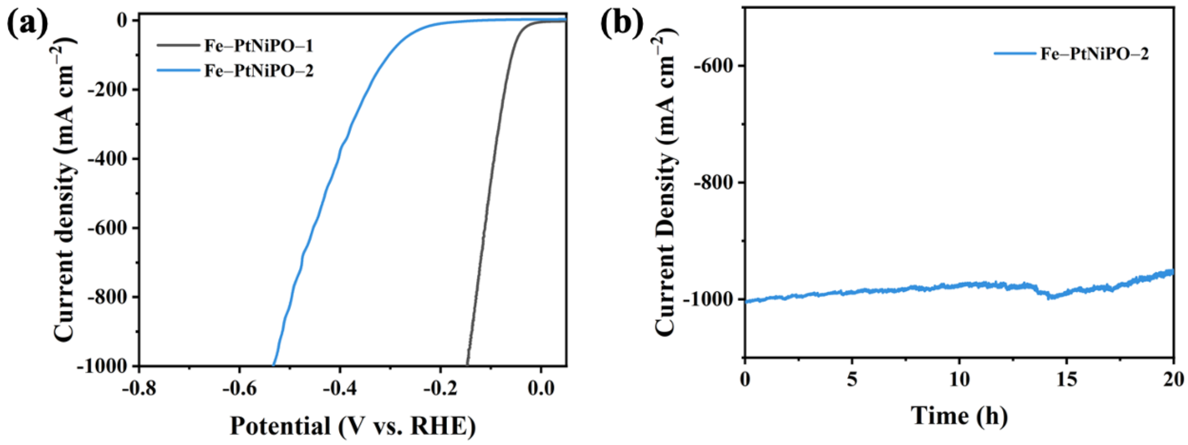

Figure S6. (a) HER polarization curves of Fe-PtNiPO-1 and Fe-PtNiPO-2. (b) Stability test of Fe-PtNiPO-2 by keeping the overpotential at 532 mV (after 90% i*R* compensation) for 20 h (with an initial current density of 1000 mA cm^{-2}).

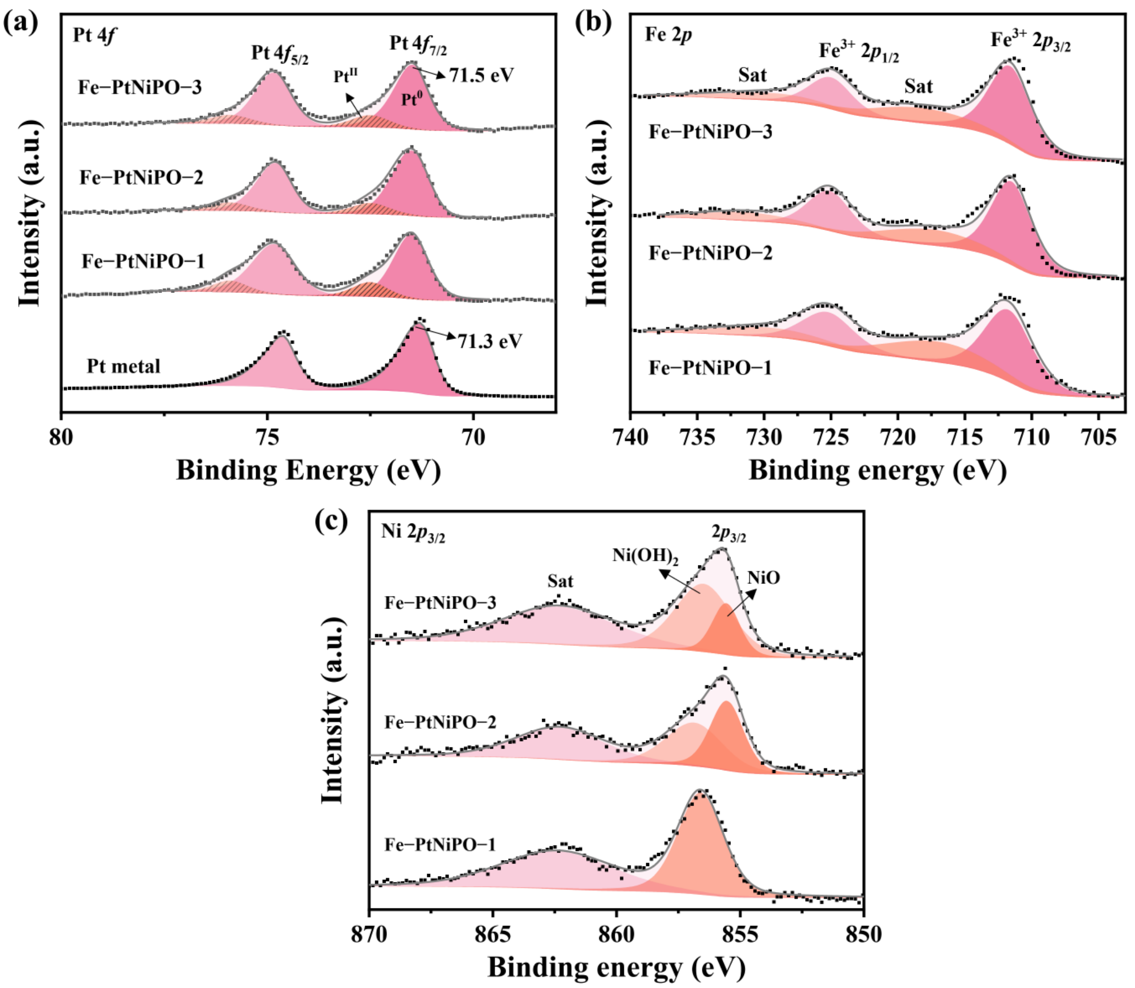

Figure S7. XPS spectra of Fe-PtNiPO-1, Fe-PtNiPO-2 and Fe-PtNiPO-3 at (a) Pt 4*f*, (b) Fe 2*p* and (c) Ni $2p_{3/2}$, respectively.

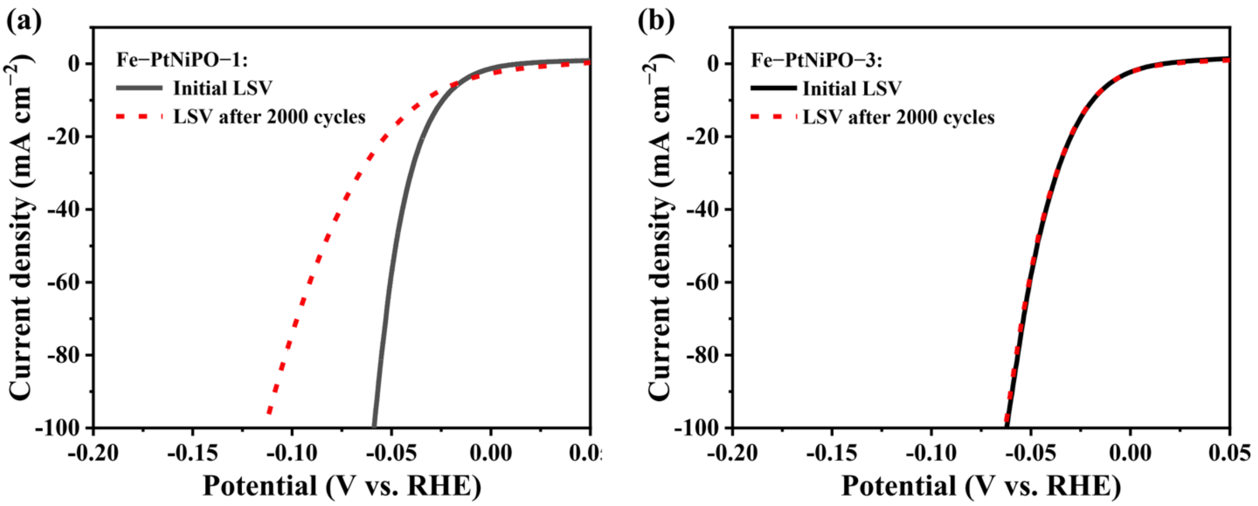

Figure S8. Catalytic durability tests of (a) Fe-PtNiPO-1 and (b) Fe-PtNiPO-3 with the initial polarization curve and the curve after 2000 cycles (dashed line) (after 90% i*R* compensation).

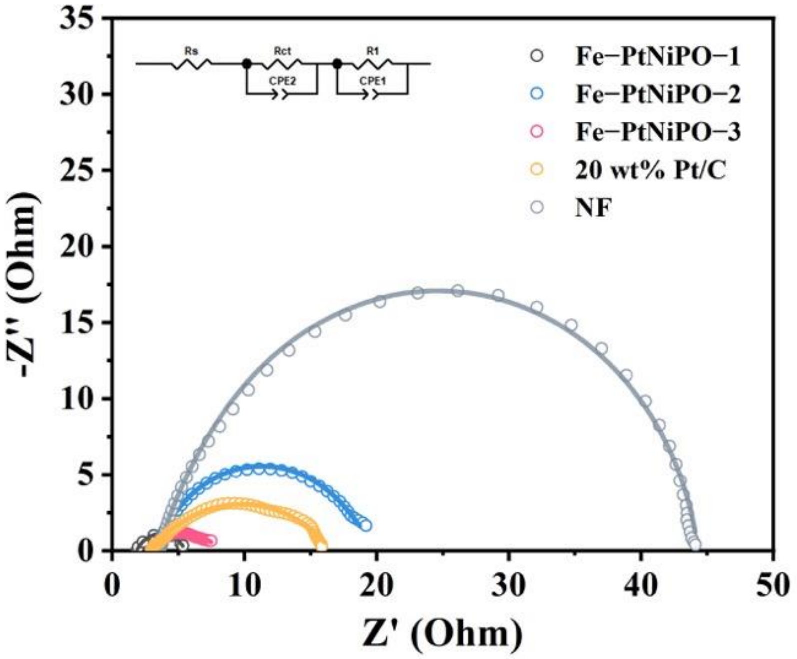

Figure S9. Electrochemical impedance spectra of Fe-PtNiPO-1, Fe-PtNiPO-2, Fe-PtNiPO-3, 20 wt% Pt/C and NF in 1.0 M KOH.

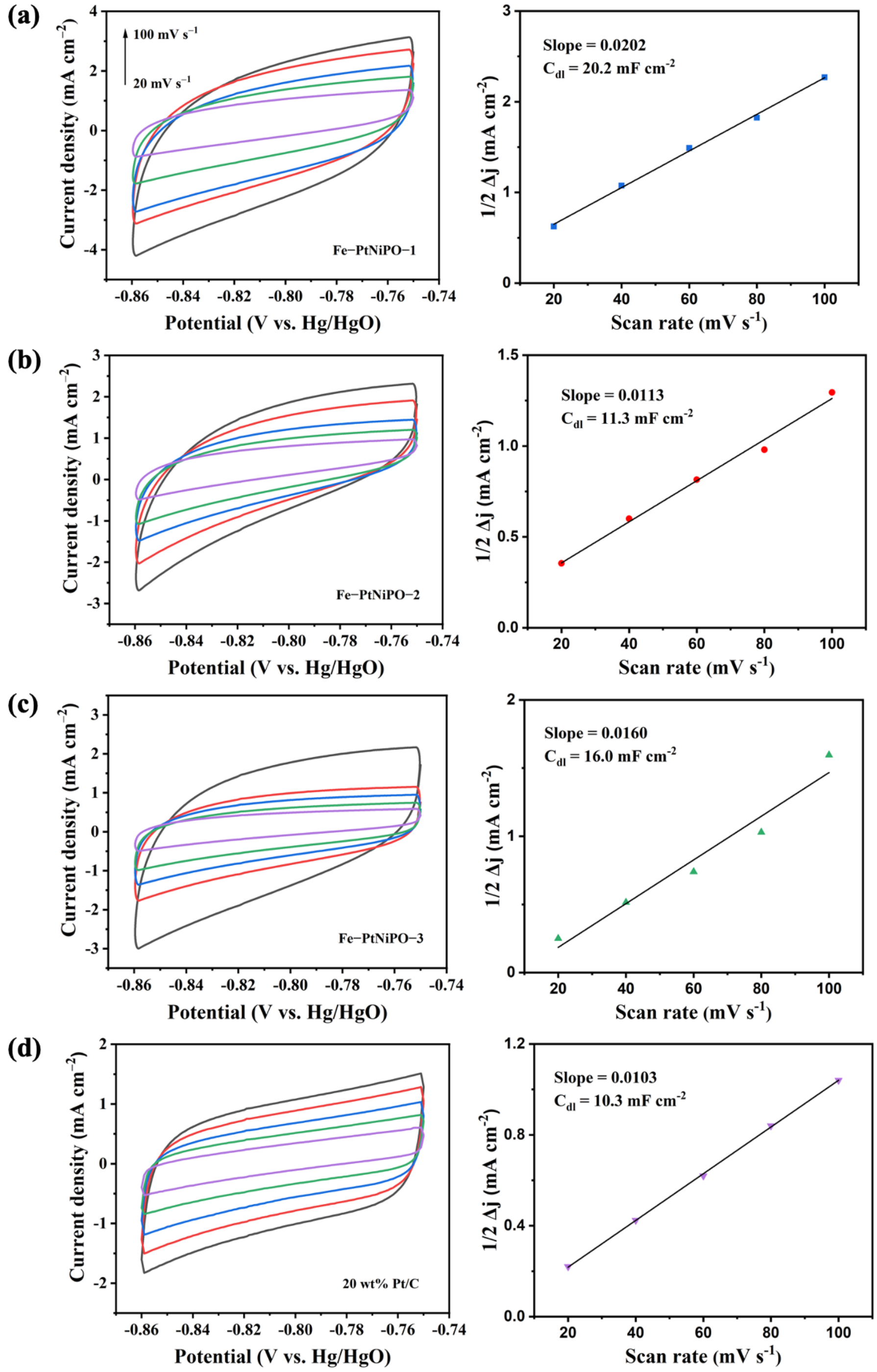

Figure S10. CV curves at different scan rates and the calculated C_{dl} for various catalysts. (a) Fe-PtNiPO-1, (b) Fe-PtNiPO-2, (c) Fe-PtNiPO-3 and (d) 20 wt% Pt/C in 1.0 M KOH.

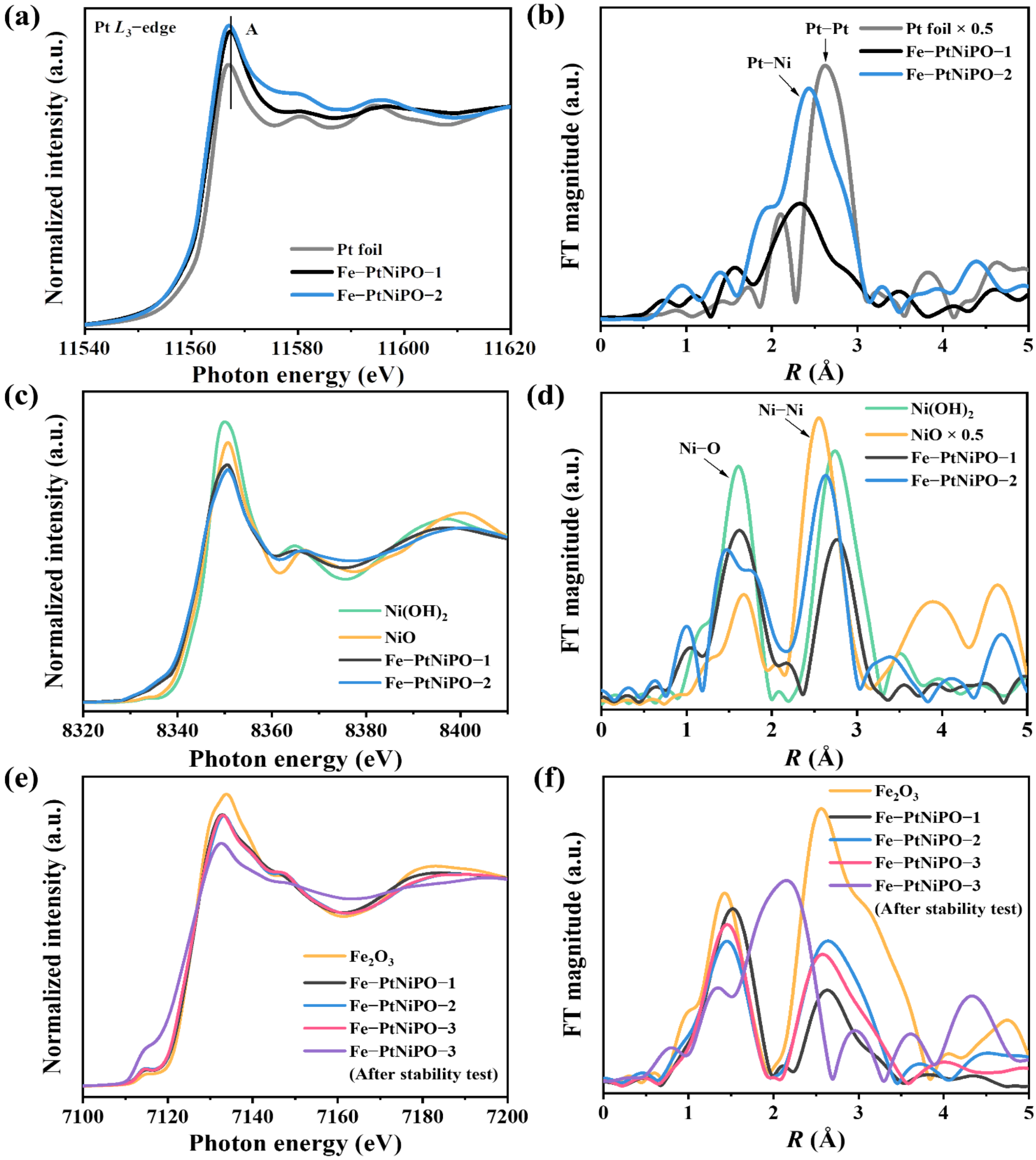

Figure S11. (a) XANES spectra of Pt foil, Fe-PtNiPO-1, and Fe-PtNiPO-2 at Pt L_3-edge. (c) XANES spectra of $Ni(OH)_2$, NiO, Fe-PtNiPO-1, Fe-PtNiPO-2 at Ni *K*-edge. (e) XANES spectra of Fe_2O_3, Fe-PtNiPO-1, Fe-PtNiPO-2, Fe-PtNiPO-3 and Fe-PtNiPO-3 (After stability test) at Fe *K*-edge. (b, d, f) Corresponding Fourier transform curves of the EXAFS data.

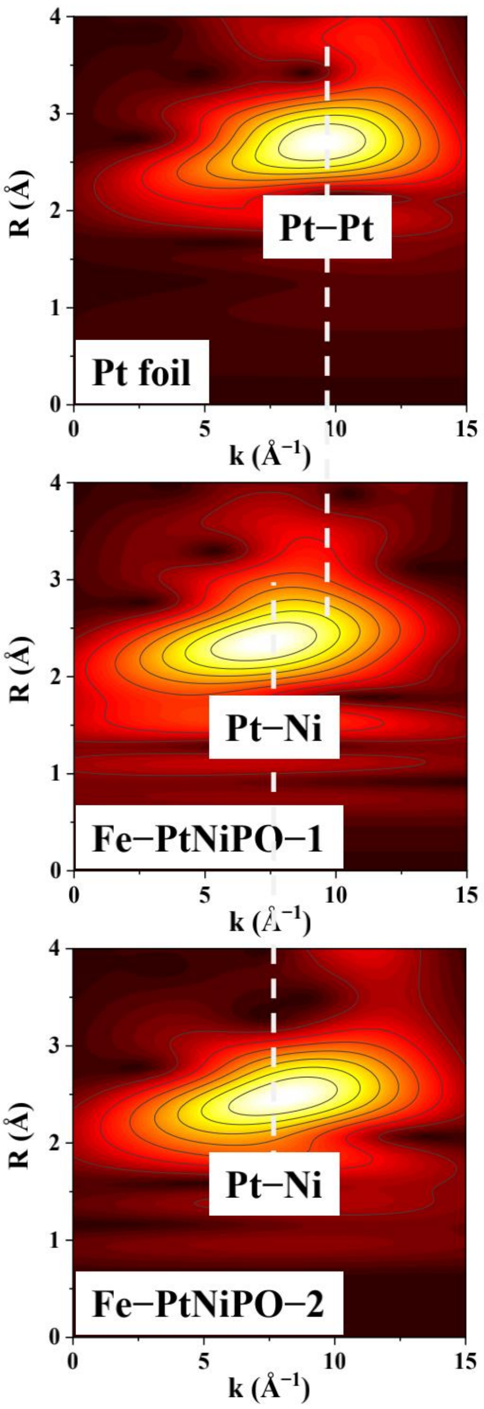

Figure S12. WT plots of the EXAFS data for Pt foil, Fe-PtNiPO-1 and Fe-PtNiPO-2, showing the *k* and *R* space data simultaneously.

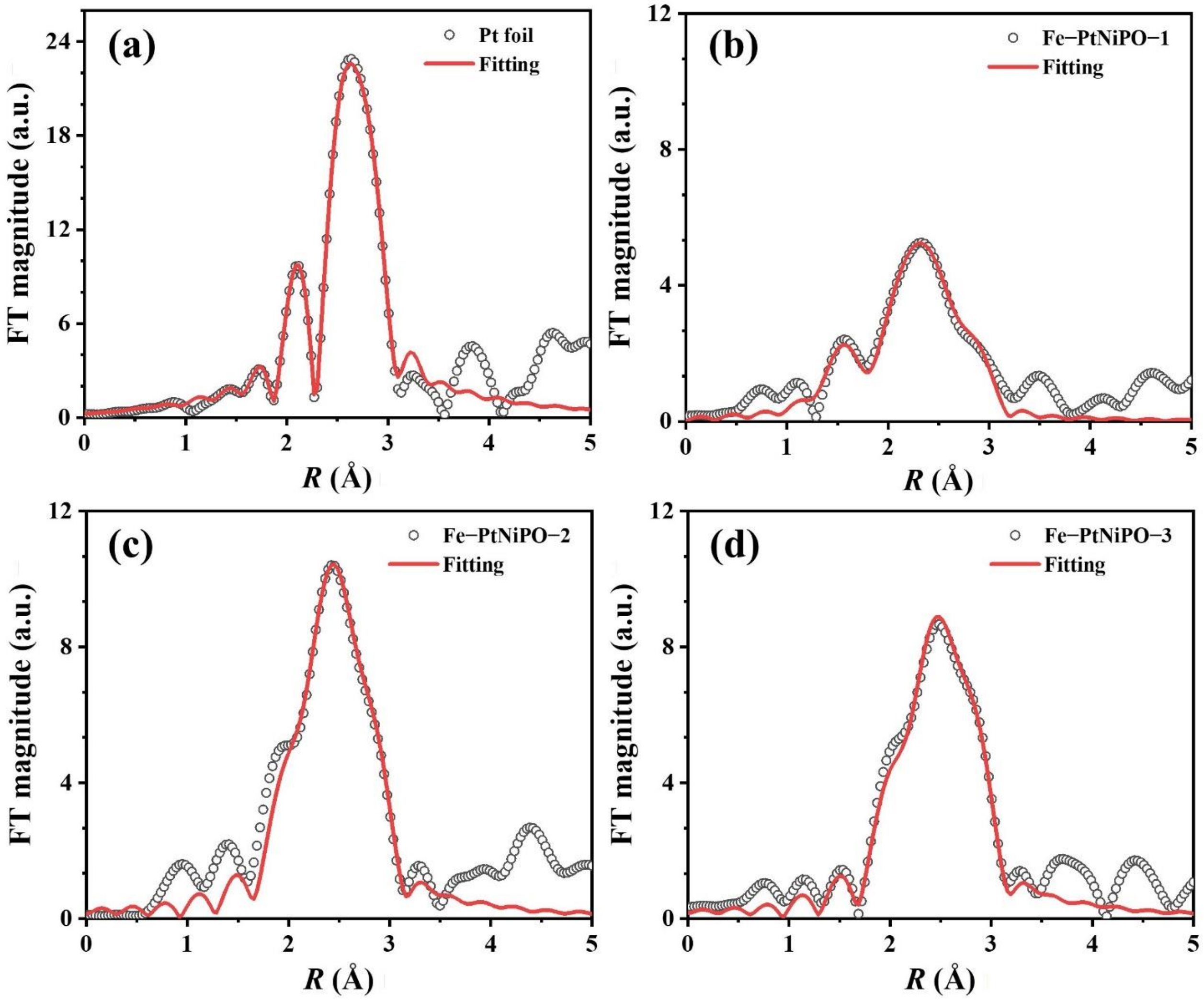

Figure S13. Fourier transform and fitting curves of the EXAFS data at Pt L_3-edge for (a) Pt foil, (b) Fe-PtNiPO-1, (c) Fe-PtNiPO-2 and (d) Fe-PtNiPO-3, respectively.

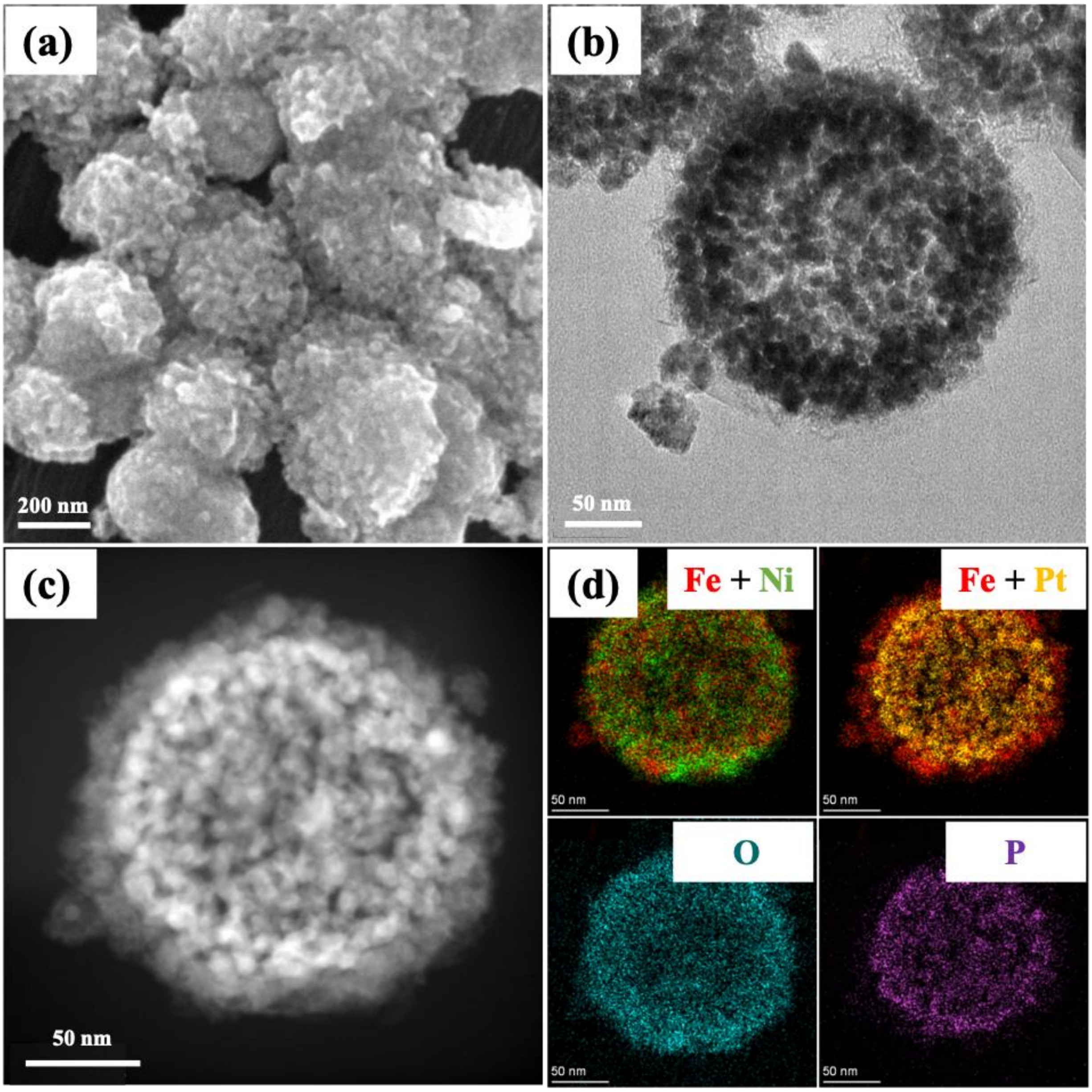

Figure S14. (a) SEM and (b) TEM images of Fe-PtNiPO-3 (after stability test). (c) HAADF-STEM image and (d) the corresponding dark-field elemental mappings of Fe-PtNiPO-3 (after stability test). Fe, Ni, Pt, P and O are represented as red, green, yellow, purple and cyan, respectively.

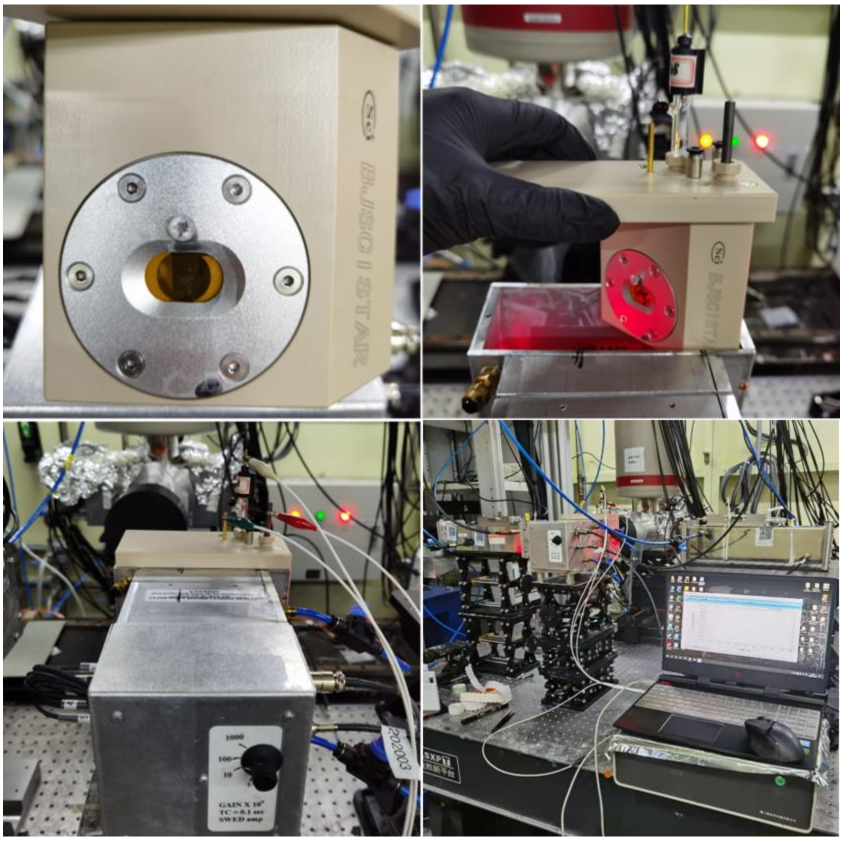

Figure S15. Experimental set up for the *in-situ* XAS experiments.

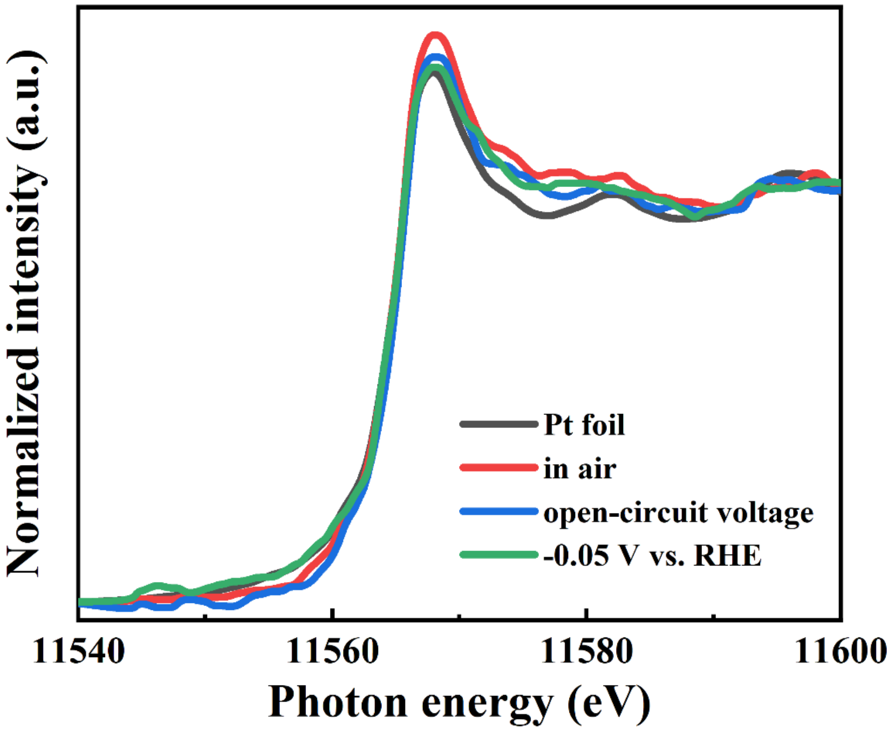

Figure S16. *In-situ* XANES spectra of Fe-PtNiPO-3 at different reaction stages at Pt L_3-edge.

Table S1. ICP data of Fe-PtNiPO-1, Fe-PtNiPO-2, Fe-PtNiPO-3, Fe-PtNiPO-3 after stability test and 20 wt% Pt/C.#

Samples	Weight (wt%)		
	Pt	Fe	P
Fe-PtNiPO-1	0.14	0.41	0.02
Fe-PtNiPO-2	0.08	0.35	0.01
Fe-PtNiPO-3	0.09	0.31	0.01
Fe-PtNiPO-3 after stability test	0.09	0.21	~0
20 wt% Pt/C	0.29	—	—

All the catalysts are measured on NF.

Table S2. Comparison of the electrocatalytic performance of Fe-PtNiPO-3 with some representative electrodes (or electrocatalysts) reported for HER in the alkaline environment.

Catalyst	Overpotential @ Current density (mV)		Stability/h	Ref.
	10 mA cm^{-2}	High current density		
Fe-PtNiPO-3	19	193 @ 1000	300 (1000 mA cm^{-2})	This work
V_O-Ru/HfO_2-OP	29	-	30 (10 mA cm^{-2})	1
Pt_3Co@NCNT	36	-	10 (100 mA cm^{-2})	2
B-Os aerogels	19	-	20 (10 mA cm^{-2})	3
Pt_{SA}-NiO/Ni	26	85 @ 100	30 (20 mA cm^{-2})	4
$NiRu_{0.13}$-BDC	34	-	-	5
Ru/np-MoS_2	30	-	40 (10 mA cm^{-2})	6
Pt-SAs/$MoSe_2$	29	-	-	7
$PtTe_2$-600 NSs	22	-	24 (200 mA cm^{-2})	8
Pt_1/N-C	46	-	20 (10 mA cm^{-2})	9
C-Ir_1/$Co_{0.8}Fe_{0.2}Se_2$@NF	4	-	100 (10 mA cm^{-2})	10
$SrTi_{0.7}Ru_{0.3}O_{3-\delta}$	46	-	200 (10 mA cm^{-2})	11
Ru-NC-700	12	-	-	12
Ru@C_2N	17	-	-	13
#RuCo alloy-nanosheets	10	54 @ 100	50 (320 mA cm^{-2})	14

MoC–Mo_2C-790	98.2	292 @ 500	50 (500 mA cm^{-2})	15
MoS_2/Mo_2C	-	220 @ 1000	24 (200 mA cm^{-2})	16
HC-MoS_2/Mo_2C	-	412 @ 1000	24 (400 mA cm^{-2})	17
Ni_2P/NF	-	306 @ 1000	10 (2500 mA cm^{-2})	18
P-Fe_3O_4/IF	-	~240 @ 1000	1000 (1000 mA cm^{-2})	19
A-NiCo LDH/NF	36	381 @ 1000	72 (1000 mA cm^{-2})	20
RFNOH-10	13	152 @ 1000	20 (500 mA cm^{-2})	21
NiMnOP/NF	91	195 @ 500	35 (500 mA cm^{-2})	22
$NiMoO_x$/NiMoS	38	236 @ 1000	25 (500 mA cm^{-2})	23
Ru-CoO_x/NF	20	252 @ 1000	100 (~70 mA cm^{-2})	24
#Ni-P-B/Ni foam	-	~275 @ 1000	240 (1000 mA cm^{-2})	25
Mo-/Co-N-C/Cu	22	230 @ 1000	200 (50 mA cm^{-2})	26
Co-Mo_5N_6	19	280 @ 1000	10 (1000 mA cm^{-2})	27
NiP_2-FeP_2/Cu_{NW}/Cu_f	23.6	357 @ 1000	50 (1000 mA cm^{-2})	28
Co/Se-MoS_2-NF	-	382 @ 1000	350 (1000 mA cm^{-2})	29

Note: #sample represents the overpotential @ current density is estimated from the HER polarization curve shown in the paper.

References

1 Li, G. *et al.* The synergistic effect of Hf-O-Ru bonds and oxygen vacancies in Ru/HfO_2 for enhanced hydrogen evolution. *Nat. Commun.* **13**, 1270 (2022).

2 Zhang, S. L., Lu, X. F., Wu, Z. P., Luan, D. & Lou, X. W. D. Engineering Platinum-Cobalt Nano-alloys in Porous Nitrogen-Doped Carbon Nanotubes for Highly Efficient Electrocatalytic Hydrogen Evolution. *Angew. Chem. Int. Ed.* **60**, 19068-19073 (2021).

3 Li, Y. *et al.* Interstitial boron-triggered electron-deficient Os aerogels for enhanced pH-universal hydrogen evolution. *Nat. Commun.* **13**, 1143 (2022).

4 Zhou, K. L. *et al.* Platinum single-atom catalyst coupled with transition metal/metal oxide heterostructure for accelerating alkaline hydrogen evolution reaction. *Nat. Commun.* **12**, 3783 (2021).

5 Sun, Y. *et al.* Modulating electronic structure of metal-organic frameworks by introducing atomically dispersed Ru for efficient hydrogen evolution. *Nat. Commun.* **12**, 1369 (2021).

6 Jiang, K. *et al.* Rational strain engineering of single-atom ruthenium on nanoporous MoS_2 for highly efficient hydrogen evolution. *Nat. Commun.* **12**, 1687 (2021).

7 Shi, Y. *et al.* Electronic metal-support interaction modulates single-atom platinum catalysis for hydrogen evolution reaction. *Nat. Commun.* **12**, 3021 (2021).

8 Li, X. *et al.* Ordered clustering of single atomic Te vacancies in atomically thin $PtTe_2$ promotes hydrogen evolution catalysis. *Nat. Commun.* **12**, 2351 (2021).

9 Fang, S. *et al.* Uncovering near-free platinum single-atom dynamics during electrochemical hydrogen evolution reaction. *Nat. Commun.* **11**, 1029 (2020).

10 Zhang, Z. *et al.* Electrochemical deposition as a universal route for fabricating single-atom catalysts. *Nat. Commun.* **11**, 1215 (2020).

11 Dai, J. *et al.* Single-phase perovskite oxide with super-exchange induced atomic-scale synergistic active centers enables ultrafast hydrogen evolution. *Nat. Commun.* **11**, 5657 (2020).

12 Lu, B. *et al.* Ruthenium atomically dispersed in carbon outperforms platinum toward hydrogen evolution in alkaline media. *Nat. Commun.* **10**, 631 (2019).

13 Mahmood, J. *et al.* An efficient and pH-universal ruthenium-based catalyst for the hydrogen evolution reaction. *Nat. Nanotechnol.* **12**, 441-446 (2017).

14 Cai, C. *et al.* Optimizing Hydrogen Binding on Ru Sites with RuCo Alloy Nanosheets for

Efficient Alkaline Hydrogen Evolution. *Angew. Chem. Int. Ed.* **61**, 202113664 (2022).

15 Liu, W. *et al.* A durable and pH-universal self-standing MoC-Mo_2C heterojunction electrode for efficient hydrogen evolution reaction. *Nat. Commun.* **12**, 6776 (2021).

16 Luo, Y. *et al.* Morphology and surface chemistry engineering toward pH-universal catalysts for hydrogen evolution at high current density. *Nat. Commun.* **10**, 269 (2019).

17 Zhang, C. *et al.* High-throughput production of cheap mineral-based two-dimensional electrocatalysts for high-current-density hydrogen evolution. *Nat. Commun.* **11**, 3724 (2020).

18 Yu, X. *et al.* "Superaerophobic" Nickel Phosphide Nanoarray Catalyst for Efficient Hydrogen Evolution at Ultrahigh Current Densities. *J. Am. Chem. Soc.* **141**, 7537-7543 (2019).

19 Zhang, J. *et al.* Modulation of Inverse Spinel Fe_3O_4 by Phosphorus Doping as an Industrially Promising Electrocatalyst for Hydrogen Evolution. *Adv. Mater.* **31**, 1905107 (2019).

20 Yang, H., Chen, Z., Guo, P., Fei, B. & Wu, R. B-doping-induced amorphization of LDH for large-current-density hydrogen evolution reaction. *Appl. Catal. B-Environ* **261**, 118240 (2020).

21 Xiao, X. *et al.* In Situ Growth of Ru Nanoparticles on (Fe,Ni)$(OH)_2$ to Boost Hydrogen Evolution Activity at High Current Density in Alkaline Media. *Small Methods* **4**, 1900796 (2020).

22 Balamurugan, J., Nguyen, T. T., Aravindan, V., Kim, N. H. & Lee, J. H. Highly reversible water splitting cell building from hierarchical 3D nickel manganese oxyphosphide nanosheets. *Nano Energy* **69**, 104432 (2020).

23 Zhai, P. *et al.* Engineering active sites on hierarchical transition bimetal oxides/sulfides heterostructure array enabling robust overall water splitting. *Nat. Commun.* **11**, 5462 (2020).

24 Wu, D., Chen, D., Zhu, J. & Mu, S. Ultralow Ru Incorporated Amorphous Cobalt-Based Oxides for High-Current-Density Overall Water Splitting in Alkaline and Seawater Media. *Small* **17**, 2102777 (2021).

25 Hao, W. *et al.* Fabrication of practical catalytic electrodes using insulating and eco-friendly substrates for overall water splitting. *Energy Environ. Sci.* **13**, 102-110 (2020).

26 Shi, H. *et al.* Mo-/Co-N-C Hybrid Nanosheets Oriented on Hierarchical Nanoporous Cu as Versatile Electrocatalysts for Efficient Water Splitting. *Adv. Funct. Mater.* **31**, 2102285 (2021).

27 Lin, F. *et al.* Electrocatalytic Hydrogen Evolution of Ultrathin Co-Mo_5N_6 Heterojunction with Interfacial Electron Redistribution. *Adv. Energy Mater.* **10**, 2002176 (2020).

28 Kumar, A. *et al.* Modulating Interfacial Charge Density of NiP_2–FeP_2 via Coupling with Metallic Cu for Accelerating Alkaline Hydrogen Evolution. *ACS Energy Lett.* **6**, 354-363 (2021).

29 Zheng, Z. *et al.* Boosting hydrogen evolution on MoS_2 via co-confining selenium in surface and cobalt in inner layer. *Nat. Commun.* **11**, 3315 (2020).

Table S3. Simulation results of R_{ct} and R_s for various samples (Data derived from electrochemical impedance spectra).

Samples	R_{ct} (Ω)	R_s (Ω)
Fe-PtNiPO-1	3.7	1.9
Fe-PtNiPO-2	15.7	3.3
Fe-PtNiPO-3	4.3	2.9
20 wt% Pt/C	13.8	2.6
NF	41.4	3.5

Table S4. Structure parameters extracted from the EXAFS fitting at Pt L_3-edge[a].

Samples	Path	S_0^2	CN	R (Å)	ΔE_0 (eV)	σ^2 (10^{-3} Å^2)
Pt foil	Pt-Pt	0.82	12	2.76	7.6	4.50
Fe-PtNiPO-1	Pt-O	0.82	0.5	1.97	4.9	4.97
	Pt-Ni	0.82	2.7	2.64	4.9	10.17
	Pt-Pt	0.82	6.9	2.75	4.9	10.90
Fe-PtNiPO-2	Pt-O	0.82	0.2	1.94	5.7	10.30
	Pt-Ni	0.82	4.9	2.65	5.7	11.96
	Pt-Pt	0.82	8.6	2.78	5.7	6.89
Fe-PtNiPO-3	Pt-O	0.82	0.3	1.95	5.0	10.27
	Pt-Ni	0.82	3.2	2.65	5.0	9.57
	Pt-Pt	0.82	8.4	2.76	5.0	6.56

Note. [a] CN is the coordination number; R is the interatomic distance; ΔE_0 is the edge-energy shift (difference between the zero kinetic energy value of the sample and that of the theoretical model); σ^2 is the Debye-Waller factor.
